# Supplementary material for: Humanity’s diverse predatory niche and its ecological consequences
Source: Commun Biol. 2023 Jun 29;6:609. doi: 10.1038/s42003-023-04940-w (PMC10310721; doi:10.1038/s42003-023-04940-w)
Supplement: Supplementary file 2 — Supplementary Information [file 42003_2023_4940_MOESM2_ESM.pdf]

Supplementary information for:

Humanity's diverse predatory niche and its ecological consequences

Chris T. Darimont[1,2]* Rob Cooke[3*], Mathieu L. Bourbonnais[4], Heather M. Bryan[2,5], Stephanie M. Carlson[6], James A. Estes[7], Mauro Galetti[8,9], Taal Levi[10], Jessica L. Maclean[1,2], Iain McKechnie[11,12], Paul C. Paquet[1,2], Boris Worm[13,14]

Affiliations:

[1]Department of Geography, University of Victoria; Victoria, Canada

[2]Raincoast Conservation Foundation; Sidney, Canada

[3]UK Centre for Ecology & Hydrology; Wallingford, UK

[4]Department of Earth, Environmental, and Geographic Sciences, University of British Columbia Okanagan; Kelowna, Canada

[5]Department of Ecosystem Science and Management, University of Northern British Columbia; Prince George, Canada

[6]Department of Environmental Science, Policy, and Management, University of California; Berkeley, USA

[7]Department of Ecology and Evolutionary Biology, University of California; Santa Cruz, USA

[8] São Paulo State University (UNESP), Department of Biodiversity, Rio Claro, SP, Brazil

[9] Kimberly Green Latin American and Caribbean Center, Florida International University (FIU), Miami, FL, USA

[10]Department of Fisheries, Wildlife, and Conservation Sciences, Oregon State University; Corvallis, USA

[11]Department of Anthropology, University of Victoria; Victoria, BC Canada

[12]Hakai Institute, Heriot Bay, Quadra Island; BC Canada

[13]Department of Biology, Dalhousie University; Halifax, Canada

[14]Ocean Frontier Institute, Dalhousie University; Halifax, Canada

*= these authors contributed equally.

Emails: darimont@uvic.ca; RobOke@ceh.ac.uk.

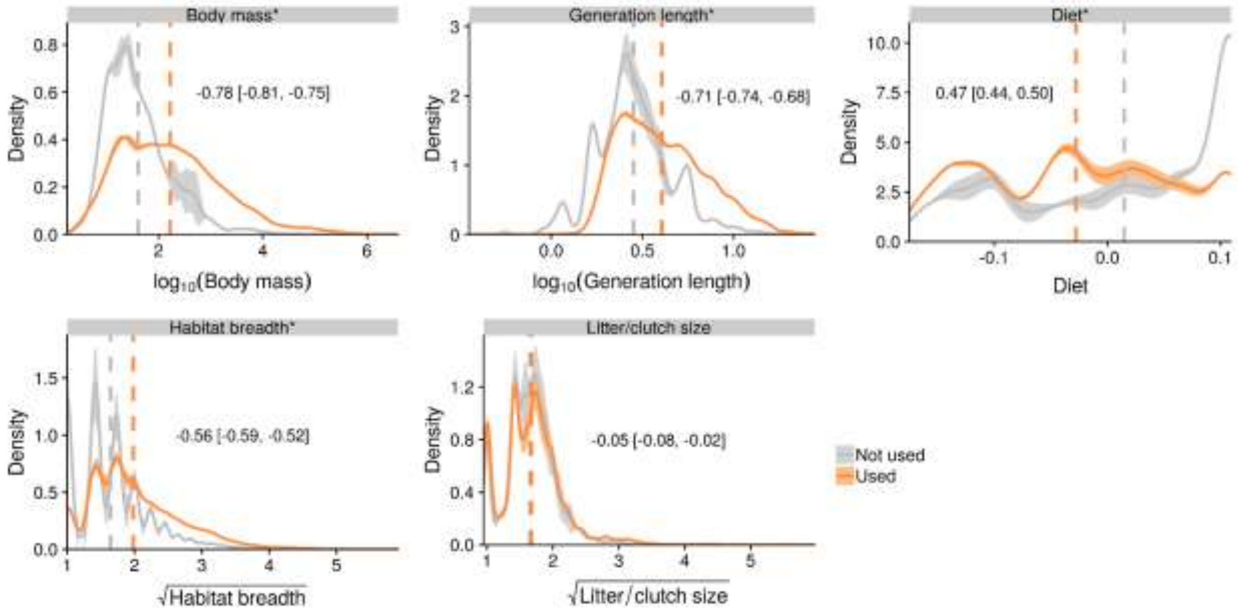

Supplementary Figure 1. Individual trait distributions. Trait distributions across those terrestrial bird and mammal species not used (10,169 species; gray) and used (6,244 species; orange) by humans. Lines show the density distribution of the traits for each group with 95% confidence envelopes (imputation uncertainty). Dashed vertical lines indicate the mean of each distribution. Labelled are the relative effect sizes of the differences between the distributions with 95% confidence intervals. Asterisks indicate the traits with statistically different distributions ($P \leq 0.05$), based on Mann-Whitney U tests (two-sided). Body mass: W = 19,408,512, n_1 = 10,169, n_2 = 6,244, P = ~0; Generation length: W = 20,245,963, n_1 = 10,169, n_2 = 6,244, P = ~0; Diet: W = 40,984,746, n_1 = 10,169, n_2 = 6,244, P = ~0; Habitat breadth: W = 22,234,960, n_1 = 10,169, n_2 = 6,244, P = ~0; Litter/clutch size: W = 31,489,292, n_1 = 10,169, n_2 = 6,244, P = 0.42.

Supplementary Table 1. IUCN Use and Trade Classification – Version 1.0.

ID Number	End Use	Referred to in-text
1	Food – human	Food
2	Food – animal	Animal feed
3	Medicine - human & veterinary	Medicine
4	Poisons	Poisons
5	Manufacturing chemicals	
6	Other chemicals	
7	Fuels	
8	Fibre	
9	Construction or structural	
10	Wearing apparel, accessories	Clothes
11	Other household goods	
12	Handicrafts, jewelry, etc.	
13	Pets/display animals, horticulture	Pets
14	Research	
15	Sport hunting/specimen collecting	Collection
16	Establish ex-situ production	
17	Other	
18	Unknown	

Supplementary Table 2. Species identities depicted in Figure 2.

Panel	Species	Common Name	Class	IUCN risk status	Realm
c	*Panthera leo*	African lion	Mammalia	Vulnerable	Terrestrial
d	*Salvelinus alpinus*	Arctic char	Actinopterygii	Least Concern	Freshwater
e	*Thunnus thynnus*	Atlantic bluefin tuna	Actinopterygii	Endangered	Marine
f	*Thalurania glaucopis*	Violet-capped woodnymph	Aves	Least Concern	Terrestrial
g	*Crocodylus acutus*	American crocodile	Reptilia	Vulnerable	Terrestrial, Marine, Freshwater
h	*Chaetodon rainfordi*	Rainford's butterflyfish	Actinopterygii	Near Threatened	Marine
i	*Manis culionensis*	Philippine pangolin	Mammalia	Critically Endangered	Terrestrial
J	*Ceratotherium simum cottoni*	Northern White rhinoceros	Mammalia	Critically Endangered	Terrestrial
k	*Ursus thibetanus*	Asiatic black bear	Mammalia	Vulnerable	Terrestrial
l	*Prionace glauca*	Blue shark	Chondrichthyes	Near Threatened	Marine
m	*Bison bison*	American bison	Mammalia	Near Threatened	Terrestrial
n	*Phyllobates terribilis*	Golden poison frog	Amphibia	Endangered	Terrestrial, Freshwater
o	*Oncorhynchus nerka*	Sockeye salmon	Actinopterygii	Least Concern	Freshwater, Marine
P	*Trimeresurus albolabris*	White-lipped Tree viper	Reptilia	Least Concern	Terrestrial
q	*Pharomachrus mocinno*	Resplendent quetzel	Aves	Near Threatened	Terrestrial
r	*Rhinoplax vigil*	Helmeted hornbill	Aves	Critically Endangered	Terrestrial
s	*Macaca mulatta*	Rhesus macaque	Mammalia	Least Concern	Terrestrial
