# Supplementary material for: Humanity’s diverse predatory niche and its ecological consequences
Source: Commun Biol. 2023 Jun 29;6:609. doi: 10.1038/s42003-023-04940-w (PMC10310721; doi:10.1038/s42003-023-04940-w)
Supplement: Supplementary file 3 — Reporting Summary [file 42003_2023_4940_MOESM3_ESM.pdf]

Corresponding author(s): Chris Darimont and Rob Cooke

Last updated by author(s): Feb 13, 2023

# Reporting Summary

Nature Portfolio wishes to improve the reproducibility of the work that we publish. This form provides structure for consistency and transparency in reporting. For further information on Nature Portfolio policies, see our Editorial Policies and the Editorial Policy Checklist.

## Statistics

For all statistical analyses, confirm that the following items are present in the figure legend, table legend, main text, or Methods section.

| n/a | Confirmed | |
|---|---|---|
| ☐ | ☒ | The exact sample size (*n*) for each experimental group/condition, given as a discrete number and unit of measurement |
| ☐ | ☒ | A statement on whether measurements were taken from distinct samples or whether the same sample was measured repeatedly |
| ☐ | ☒ | The statistical test(s) used AND whether they are one- or two-sided<br>*Only common tests should be described solely by name; describe more complex techniques in the Methods section.* |
| ☒ | ☐ | A description of all covariates tested |
| ☒ | ☐ | A description of any assumptions or corrections, such as tests of normality and adjustment for multiple comparisons |
| ☒ | ☐ | A full description of the statistical parameters including central tendency (e.g. means) or other basic estimates (e.g. regression coefficient) AND variation (e.g. standard deviation) or associated estimates of uncertainty (e.g. confidence intervals) |
| ☐ | ☒ | For null hypothesis testing, the test statistic (e.g. *F*, *t*, *r*) with confidence intervals, effect sizes, degrees of freedom and *P* value noted<br>*Give P values as exact values whenever suitable.* |
| ☒ | ☐ | For Bayesian analysis, information on the choice of priors and Markov chain Monte Carlo settings |
| ☒ | ☐ | For hierarchical and complex designs, identification of the appropriate level for tests and full reporting of outcomes |
| ☐ | ☒ | Estimates of effect sizes (e.g. Cohen's *d*, Pearson's *r*), indicating how they were calculated |

*Our web collection on statistics for biologists contains articles on many of the points above.*

## Software and code

Policy information about availability of computer code

| | |
|---|---|
| Data collection | Data were collected using APIv4. |
| Data analysis | All code for data analysis was written in R using versions up to 4.0.4. All code was tested for compatibility on R version 4.0.4. The code written to perform the data analyzes are available at: https://github.com/ACSLabUVic/diverse-predatory-niche. All R packages used to perform the analyzes are listed within the code, with information on the version of the packages used stored in a renv, and where applicable packages are referenced within the main text. The full list of packages used is: DBI 1.1.3, FNN 1.1.3.1, GenSA 1.1.7, KernSmooth 2.23-18, MASS 7.3-53, Matrix 1.3-2, ModelMetrics 1.2.2.2, R6 2.5.1, RColorBrewer 1.1-3, Rcpp 1.0.9, RcppArmadillo 0.11.4.2.1, RcppProgress 0.4.2, SQUAREM 2021.1, XML 3.99-0.13, abind 1.4-5, ade4 1.7-20, askpass 1.1, assertthat 0.2.1, backports 1.4.1, base64enc 0.1-3, bit 4.0.5, bit64 4.0.5, blob 1.2.3, broom 1.0.1, bslib 0.4.1, cachem 1.0.6, callr 3.7.3, caret 6.0-93, cellranger 1.1.0, class 7.3-18, classInt 0.4-8, cli 3.4.1, clipr 0.8.0, clock 0.6.1, codetools 0.2-18, colorspace 2.0-3, commonmark 1.8.1, cowplot 1.1.1, cpp11 0.4.3, crayon 1.5.2, crosstalk 1.2.0, crul 1.3, curl 4.3.3, data.table 1.14.6, dbplyr 2.2.1, dichromat 2.0-0.1, digest 0.6.31, doParallel 1.0.17, doRNG 1.8.2, dplyr 1.0.10, dtplyr 1.2.2, e1071 1.7-12, effsize 0.8.1, ellipsis 0.3.2, eulerr 7.0.0, evaluate 0.18, fansi 1.0.3, farver 2.1.1, fastcluster 1.2.3, fastmap 1.1.0, forcats 0.5.2, foreach 1.5.2, fs 1.5.2, future 1.29.0, future.apply 1.10.0, gargle 1.2.1, generics 0.1.3, geojsonsf 2.0.3, geometries 0.2.0, geometry 0.4.6.1, gghalves 0.1.4, ggplot2 3.4.0, globals 0.16.2, glue 1.6.2, googledrive 2.0.0, googlesheets4 1.0.1, gower 1.0.0, gridBase 0.4-7, gridExtra 2.3, gtable 0.3.1, hardhat 1.2.0, haven 2.5.1, highr 0.9, hitandrun 0.5-6, hms 1.1.2, htmltools 0.5.4, htmlwidgets 1.6.0, httpcode 0.3.0, httr 1.4.4, hypervolume 3.1.0, ids 1.0.1, ipred 0.9-13, isoband 0.2.6, iterators 1.0.14, itertools 0.1-3, jpeg 0.1-10, jquerylib 0.1.4, jsonify 1.2.2, jsonlite 1.8.4, kernlab 0.9-31, knitr 1.41, ks 1.14.0, labeling 0.4.2, lattice 0.20-41, lava 1.7.0, lazyeval 0.2.2, leafem 0.2.0, leaflet 2.1.1, leaflet.providers 1.9.0, leafsync 0.1.0, lifecycle 1.0.3, linprog 0.9-4, listenv 0.8.0, lpSolve 5.6.17, lubridate 1.9.0, lwgeom 0.2-10, magic 1.6-1, magrittr 2.0.3, maps 3.4.1, markdown 1.4, mclust 6.0.0, memoise 2.0.1, mgcv 1.8-33, mime 0.12, misc3d 0.9-1, missForest 1.5, modelr 0.1.10, multicool 0.1-12, munsell 0.5.0, mvtnorm 1.1-3, nlme 3.1-152, nnet 7.3-15, numDeriv 2016.8-1.1, openssl 2.0.5, pROC 1.18.0, palmerpenguins 0.1.1, parallelly 1.32.1, pbapply 1.6-0, pdist 1.2.1, pillar 1.8.1, pixmap 0.4-12, pkgconfig 2.0.3, plot3D 1.4, plyr 1.8.8, png 0.1-8, polyclip 1.10-4, polylabelr 0.2.0, |

pracma 2.4.2, prettyunits 1.1.1, processx 3.8.0, prodlim 2019.11.13, progress 1.2.2, progressr 0.12.0, proxy 0.4-27, ps 1.7.2, purrr 0.3.5, randomForest 4.6-14, rapidjsonr 1.2.0, rappdirs 0.3.3, raster 3.6-11, rcdd 1.5, readr 2.1.3, readxl 1.4.1, recipes 1.0.3, rematch 1.0.1, rematch2 2.1.2, renv 0.16.0, reprex 2.0.2, reshape2 1.4.4, rgeos 0.5-9, rlang 1.0.6, rmarkdown 2.18, rngtools 1.5.2, rpart 4.1-15, rphylopic 0.3.0, rstudioapi 0.14, rvest 1.0.3, s2 1.1.1, sass 0.4.4, scales 1.2.1, selectr 0.4-2, sf 1.0-9, sfheaders 0.4.0, sp 1.5-1, stars 0.6-0, stringi 1.7.8, stringr 1.5.0, survival 3.2-7, sys 3.4.1, terra 1.6-47, tibble 3.1.8, tidyr 1.2.1, tidyselect 1.2.0, tidyverse 1.3.2, timeDate 4021.107, timechange 0.1.1, tinytex 0.42, tmap 3.3-3, tmaptools 3.1-1, triebeard 0.3.0, tzdb 0.3.0, units 0.8-1, urltools 1.7.3, utf8 1.2.2, uuid 1.1-0, vctrs 0.5.1, viridis 0.6.2, viridisLite 0.4.1, vroom 1.6.0, widgetframe 0.3.1, withr 2.5.0, wk 0.7.1, xfun 0.35, xml2 1.3.3, yaml 2.3.6.

For manuscripts utilizing custom algorithms or software that are central to the research but not yet described in published literature, software must be made available to editors and reviewers. We strongly encourage code deposition in a community repository (e.g. GitHub). See the Nature Portfolio guidelines for submitting code & software for further information.

## Data

Policy information about availability of data

All manuscripts must include a data availability statement. This statement should provide the following information, where applicable:

- Accession codes, unique identifiers, or web links for publicly available datasets
- A description of any restrictions on data availability
- For clinical datasets or third party data, please ensure that the statement adheres to our policy

Data and annotated code are available at https://github.com/ACSLabUVic/diverse-predatory-nicheupon publication

## Human research participants

Policy information about studies involving human research participants and Sex and Gender in Research.

| | |
|---|---|
| Reporting on sex and gender | NA |
| Population characteristics | NA |
| Recruitment | NA |
| Ethics oversight | NA |

Note that full information on the approval of the study protocol must also be provided in the manuscript.

# Field-specific reporting

Please select the one below that is the best fit for your research. If you are not sure, read the appropriate sections before making your selection.

☐ Life sciences ☐ Behavioural & social sciences ☒ Ecological, evolutionary & environmental sciences

For a reference copy of the document with all sections, see nature.com/documents/nr-reporting-summary-flat.pdf

# Ecological, evolutionary & environmental sciences study design

All studies must disclose on these points even when the disclosure is negative.

| | |
|---|---|
| Study description | We analyzed IUCN 'use and trade' data to describe the taxonomic breadth of human use of vertebrates and the proportion of used species that are threatened by extinction. We also compared human use to other wide-ranging predators, we evaluated the spatial patterns of use, and assessed the ecological breadth of use. No primary investigation was undertaken, instead we extracted/collated existing data from the IUCN Red List and from the literature. |
| Research sample | We obtained information for 46,755 vertebrate species. This is data for all known vertebrates for the vertebrate classes with the most species. We also collated information on prey species, from the literature, for a geographically and taxonomically diverse sample of wide-ranging predators. These predators are not fully comprehensive (i.e., we did not collate data for all predators of vertebrates globally) but instead reflect a trade-off between data availability (requirement for range-wide dietary data) and comparability to humans (i.e., extensive ranges and broad dietary niches comprised primarily of other vertebrates as prey). |
| Sampling strategy | All species have been assessed by the IUCN, so no sampling was performed. Sample size = population size. |
| Data collection | Data were extracted from the IUCN Red List using APIv4. |
| Timing and spatial scale | Data were extracted from the IUCN Red List during April 2019. The data are global (i.e., all known terrestrial vertebrates globally). |
| Data exclusions | We excluded vertebrate classes with < 100 species for simplicity, and these classes are more difficult to compare to the larger classes. |

| | |
|---|---|
| Reproducibility | No experimental procedures undertaken. |
| Randomization | NA |
| Blinding | NA |

Did the study involve field work? ☐ Yes ☒ No

# Reporting for specific materials, systems and methods

We require information from authors about some types of materials, experimental systems and methods used in many studies. Here, indicate whether each material, system or method listed is relevant to your study. If you are not sure if a list item applies to your research, read the appropriate section before selecting a response.

## Materials & experimental systems

| n/a | Involved in the study | |
|---|---|---|
| ☒ | ☐ | Antibodies |
| ☒ | ☐ | Eukaryotic cell lines |
| ☒ | ☐ | Palaeontology and archaeology |
| ☒ | ☐ | Animals and other organisms |
| ☒ | ☐ | Clinical data |
| ☒ | ☐ | Dual use research of concern |

## Methods

| n/a | Involved in the study | |
|---|---|---|
| ☒ | ☐ | ChIP-seq |
| ☒ | ☐ | Flow cytometry |
| ☒ | ☐ | MRI-based neuroimaging |
